# Ab Initio Dot Structures Beyond the Lewis Picture

**DOI:** 10.3390/molecules26040911

**Published:** 2021-02-09

**Authors:** Michael A. Heuer, Leonard Reuter, Arne Lüchow

**Affiliations:** Institute of Physical Chemistry, RWTH Aachen University, Landoltweg 2, 52056 Aachen, Germany; michael.heuer@rwth-aachen.de (M.A.H.); leonard.reuter@rwth-aachen.de (L.R.)

**Keywords:** probability density analysis, electronic structure, Lewis structures, chemical bonding, similarity, clustering, spin coupling

## Abstract

The empirical Lewis picture of the chemical bond dominates the view chemists have of molecules, of their stability and reactivity. Within the mathematical framework of quantum mechanics, all this chemical information is hidden in the many-particle wave function Ψ. Thus, to reveal and understand it, there is great interest in enhancing the Lewis model and connecting it to computable quantities. As has previously been shown, the Lewis picture can often be recovered from the probability density ${\left|\Psi \right|}^{2}$ with probabilities in agreement with valence bond weights: the structures appear as most likely positions in the all-electron configuration space. Here, we systematically expand this topological probability density analysis to molecules with multiple bonds and lone pairs, employing correlated Slater-Jastrow wave functions. In contrast to earlier studies, non-Lewis structures are obtained that disagree with the prevalent picture and have a potentially better predictive capability. While functional groups are still recovered with these ab initio structures, the boundary between bonds and lone pairs is mostly blurred or non-existent. In order to understand the newly found structures, the Lewis electron pairs are replaced with spin-coupled electron motifs as the fundamental electronic fragment. These electron motifs—which coincide with Lewis’ electron pairs for many single bonds—arise naturally from the generally applicable analysis presented. An attempt is made to rationalize the geometry of the newly-found structures by considering the Coulomb force and the Pauli repulsion.

## 1. Introduction

Structural formulas are such an important tool for chemists that they can justifiably be labeled the ‘script of chemistry’. They not only show the geometry of molecules but also capture parts of the electronic structure by depicting bond orders and formal charges. These sketches of the electronic properties were conceived by G.N. Lewis based on empirical observations [1] and are usually referred to as ‘dot structures’. For non-radical systems, the depiction of individual electrons as dots is usually omitted. Instead, the structures are built from electron pairs which are drawn as lines. Compared to pure dot structures, the concept of electron pairs adds information on the quantized spin: two paired electrons are spin-coupled. After the dawn of quantum mechanics, the concept of Lewis structures was taken up in valence bond (VB) theory [2,3]. Within the VB interpretation, a system is no longer described by only one dot structure, but by multiple structures with different weights.

Artmann [4] and, later, Zimmermann and Van Rysselberghe [5] were the first to investigate the maxima of the probability density ${\left|\Psi \right|}^{2}$. Note that this probability density is, in principle, measurable [6]. Based on their analyses, Linnett developed the—therefore, semi-empirical—double quartet theory [7], attributing an important role to atom-centered tetrahedrons and triangles of same-spin electrons when sketching his own spin-resolved dot structures. These early works were picked up by Scemama and coworkers, who performed a topological investigation with quantum Monte Carlo [8]. Recently, probability density analysis (PDA) has been introduced [9,10,11,12] as the all-electron equivalent of the quantum theory of atoms in molecules (QTAIM) [13,14] focusing on the one-electron density. The question arises about what can be gained from all-electron in comparison to the one-electron methods that can be broadly categorized into electron density methods (e.g., QTAIM, electron localization function (ELF) [15]) and orbital localization methods (e.g., natural bond orbital (NBO) analysis [16]). Concerning electron density methods, many-body information of a chemical system is implicitly contained in the exact ground state density [17], but its retrieval remains unclear, especially for approximate solutions. Orbital localization methods, on the other hand, lose this information due to the inherent mean-field view. In contrast, all-electron approaches offer an explicit description of many-body effects. However, this comes with the cost of additional complexity.

With PDA, ab initio dot structures are obtained as most likely electron configurations, i.e., local maxima of ${\left|\Psi \right|}^{2}$. Their number increases rapidly with O(n!), which is mainly caused by the permutational symmetry of ${\left|\Psi \right|}^{2}$. These structures often agree with Lewis structures, and their importance can be evaluated by integrating the probability density over the respective maxima’s basins of attraction [11]. The resulting probabilities, which can be obtained for any wave function, have shown to be in good agreement with the weights of VB theory [12]. As such, these dot structures depict electronic structure information hidden in the many-particle wave function in condensed form.

In this work, we describe the comprehensive clustering of the local maxima. This allows for introducing additional spin information to the representative dot structures of a cluster revealing ‘electron motifs’ as characteristic spatial arrangements of spin-coupled electrons. As such, these motifs are both a generalization of the electron pair and related to Linnett’s tetrahedrons. While the term ‘electron motif’ or ’motif’ has already been used in the literature to describe arrangements of related electrons found in the electronic structure [11,18,19,20], we propose an ab initio definition in this work. Although these structures are not intended to replace Lewis dot structures, direct insight into the electronic structure is obtained from this generally applicable approach.

By systematic investigation of correlated Slater-Jastrow wave functions of 29 molecules, we identify at most four characteristic motifs per bond accounting for >99% of the total probability. Many of these motifs cannot be reconciled with the Lewis picture of electron pairs. Whereas the individual probabilities differ significantly from those of VB or complete active space (CAS) wave functions (due to the poor treatment of static correlation), the shapes of the structures can be deemed highly accurate (due to the explicit treatment of dynamic correlation) for the chosen ansatz. Lastly, we show the interaction between motifs in acetic acid and their transferability in dehydroalanine (DHA).

It should be noted that a range of methods has been developed to also connect molecular orbital theory with the Lewis picture. These methods are mostly variants of localized molecular orbitals [21,22]: natural bond orbitals [23], quasi-atomic orbitals [24], intrinsic bond orbitals [25], and the correlation theory of the chemical bond [26,27]. Furthermore, other real-space analyses have been developed that focus on partitioning the 3-dimensional space instead of the full configuration space: QTAIM [13,14], the electron localization function [28], and the electron distribution functions [29,30]. The tiling of the many-electron wave function by Schmidt and coworkers [18] is another topological analysis of ${\left|\Psi \right|}^{2}$.

## 2. Methods

In this section, the probability density analysis is described, including the definitions of electron motifs and ab initio dot structures that have been added in this work.

### 2.1. The Probability Density

The all-electron probability density *p* is obtained as the square of the normalized 3n-dimensional electronic wave function Ψ(R) for *n* electrons: $p\left(R\right)={\left|\Psi \left(R\right)\right|}^{2}$. Its argument is an electron configuration R∈R, which is an indexed set of *n* spinpositions x with indices i∈{1⋯n}:

(1) $$R=(x_{i}).$$

A spinposition is a tuple of a position vector $r\in R^{3}$ and a magnetic spin quantum number $m_{s}\in \left\{-12,+12\right\}$:

(2) $$x=(r,m_{s}).$$

An electron configuration can be regarded as a sample point from the spatial probability distribution. Since the electrons are indistinguishable, a permutation of the indices does not alter p(R).

### 2.2. Maxima, Basins of Attraction, and Probabilities

With PDA, the local maxima $R^{*}$ of p(R) are investigated. They partition the configuration space R into their basins of attraction $\Omega_{R^{*}}$ [9,10,11,12]. As such, a maximum is a representative configuration for the entire distribution of electron configurations constituting its surrounding basin. The probability $P_{R^{*}}$ of measuring the electron configuration R in a basin of attraction of a certain maximum $R^{*}$ is obtained by

(3) $$P_{R^{*}}=\int_{\Omega_{R^{*}}}p\left(R\right)dR.$$

Note that this purely topological analysis is identical to QTAIM for one-electron systems (e.g., $H_{2}^{+}$). While numerical integration of such 3n-dimensional integrals on real-space grids becomes unfeasible already for small *n*, it is possible with Monte Carlo integration via importance sampling, which is a standard procedure in any quantum Monte Carlo code.

**Figure 1:**
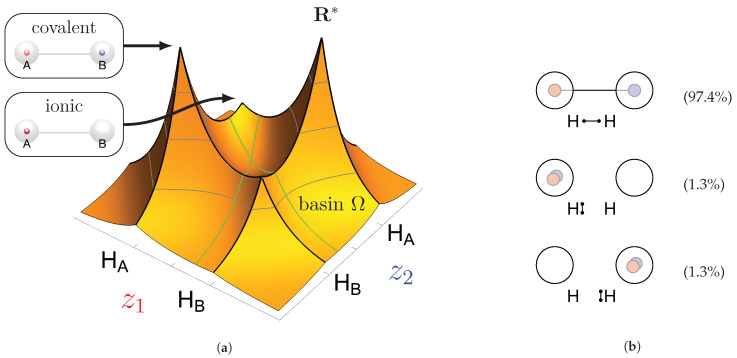
Probability density analysis for a CAS(2,2) wave function of H_2_. (**a**) Cross section of the 3n-dimensional probability density *p*(**R**) with both electrons on the z-axis. The two z-coordinates of the electrons are labeled z_1_ and z_2_ with the quantum numbers ms,1=−12 and ms,2=+12. Boundaries of the basins of attraction are indicated by green lines. (**b**) Sketches of a covalent (top) and two ionic (mid & bottom) ab initio dot structures. Cluster probabilities and corresponding Lewis structures are given.

At this point, the molecule $H_{2}$ should be briefly discussed. The protons are positioned at $\left(0,0,H_{A}\right)$ and $\left(0,0,H_{B}\right)$. The position vectors of both electrons are at these nuclei positions for all four local maxima $R^{*}$; see Figure 1a. Here, it is already apparent that the three Lewis structures of $H_{2}$ (Figure 1b) are reproduced: there are two ionic maxima (both electrons at the same proton) and two covalent maxima (one electron at each proton). Covalent and ionic maxima structures have been subject of discussion in previous works [11,31]. We label the two covalent maxima as equivalent, since they differ only in the spin coordinates and they have equal shape and value due to spin symmetry. In contrast, the ionic maxima are not labeled as equivalent, since their equal shape and value is only due to spatial symmetry. For a CAS(2,2) wave function in the minimal basis of 1s orbitals, the probabilities are 2×49% for the two covalent and 2×1.3% for the two ionic maxima [12].

**Figure A1:**
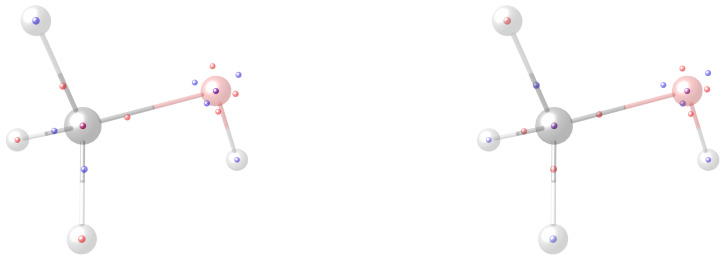
Two similar maxima of methanol. The flipped spins at the $CH_{3}$ group lead to small positional shifts of the valence electrons.

For molecules with heavier atoms, valence electrons in the maxima are no longer at core positions. While there are still equivalent maxima and qualitatively different maxima, others have very similar overall arrangements, with small displacements of the individual electrons positions (see Figure A1 in Appendix A). These small differences arise from spin permutations, the number of which increases with n! [11]. Accordingly, to allow their chemical interpretation, it is crucial to group equivalent and similar maxima into clusters.

### 2.3. Clustering of Maxima

A distance *d* between two spinpositions a and b is defined simply as the difference between the two position vectors:

(4) $$d\left(a,b\right)=|r_{a}-r_{b}|.$$

In order to compare two electron configurations $A^{\prime }$ and B, the best-match permutation (permuting $A^{\prime }$ to A) is identified [9,10] that minimizes the cost function

(5) $$\sum_{i}d\left(a_{i},b_{i}\right).$$

We then denote A as *assigned* to B. After this assignment, the distance between both configurations is calculated by the metric

(6) $$D\left(A,B\right)=\max_{i}d\left(a_{i},b_{i}\right)$$

as the maximal single-electron distance. This metric has two advantages: first, it is independent on the electron number; second, it discriminates small deviations in many electrons from a large deviation in a single electron. Note that other metric choices are possible, but their discussion exceeds the scope of this work.

A cluster C is defined as a set of assigned maxima. At the beginning, each maximum is a cluster itself. Two clusters A and B can be merged, if their distance

(7) $$D\left(A,B\right)=\min_{A\in A,B\in B}D\left(A,B\right)$$

is less than a similarity threshold $d_{\mathrm{sim}}$. This distance is the linkage function employed in single-linkage cluster analysis [32,33]. Once the distances between all pairs of remaining clusters is larger than $d_{\mathrm{sim}}$, the clustering is complete.

Then, the cluster probability of a cluster C

(8) $$P_{C}=\sum_{R^{*}\in C}P_{R^{*}}$$

is the total probability of its elements (i.e., its maxima). These probabilities compare well to the weights of valence bond theory [12].

For larger systems, it is often advantageous to restrict the elements a∈A in (5) and (6) to a subset $\tilde{A}\subset A$ (i.e., $a\in \tilde{A}$). For instance, the subset $\tilde{A}$ can be defined by the distance of its elements to the center of a bond, if only this bond is to be investigated. This yields local basin and cluster probabilities $P_{{\tilde{R}}^{*}}$ and $P_{\tilde{C}}$, respectively. By means of this ‘local clustering’, the combinatorial explosion of the number of clusters for large molecules is tamed.

If the four maxima of $H_{2}$ (Figure 1a) are clustered with this metric D and a threshold less than the proton-proton distance (i.e., $d_{\mathrm{sim}}<d($HA, HB)), the two covalent maxima are clustered together, while the two ionic maxima remain separated. With this approach, the three Lewis structures are reproduced; see Figure 1b.

**Figure 2:**
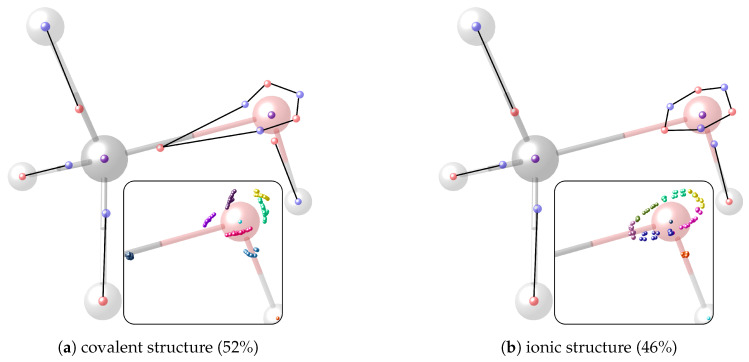
Representative maxima of the two most probable clusters in methanol and their cluster probabilities $P_{C}$ obtained for $$d_{\mathrm{sim}}=0.2a_{0}$$. *α* and *β* electrons are visualized in semi-transparent red and blue so that coinciding opposite-spin electrons appear as violet. Black lines connect spin-coupled electrons $c_{ij}^{C}=-1$ forming electron motifs. The nucleus positions are represented as transparent spheres, bond lines are added for clarity. The insets show the overlay of all assigned maxima of the respective cluster. Electrons with the same index have the same color.

For methanol and a threshold of $d_{\mathrm{sim}}=0.2a_{0}$ (Bohr radius), two significant clusters are obtained that are depicted in Figure 2.

Their probabilities sum up to 98%. The remaining 2% are constituted by several minor clusters with low probabilities showing the same motifs at the C−O and O−H bonds but with an ionic motif at one or multiple C−H bonds. If only the C−O bond is of interest, local cluster probabilities of 53% for the covalent and 47% for the ionic cluster are obtained.

The insets in the figures show all maxima within the covalent (Figure 2a) and the ionic cluster (Figure 2b). In these insets, the electrons are color-coded according by their index and show the positional spread of the maxima, which is a result of opposite-spin permutations (cf. Figure A1 in Appendix A). It is apparent that the positional differences of the lone pair electrons across the maxima of one cluster are much larger than for the bond electrons, which has already been discussed in previous work for methylamine [11]. Still, all maxima within one cluster are structurally similar.

Based on these observations and with the goal of keeping the overall bias introduced to our analysis minimal, we adhere to the following guideline for the choice of $d_{\mathrm{sim}}$, the single parameter of the proposed clustering: choose $d_{\mathrm{sim}}$ so that maxima with different formal charges (i.e., different number of electrons in the vicinity of atoms) are separated but structurally similar maxima originating solely from spin-permutations are clustered together.

Next, we utilize the spin information contained in the clustered maxima. This will lead to the recovery of the lines connecting the dots in the Lewis structures and beyond.

### 2.4. Spin Correlation, Electron Motifs, and Ab Initio Dot Structures

After the maxima clustering, a representative maximum $R_{C}^{*}$ can be chosen for each cluster (e.g., the one with the highest function value p(R)). Additionally, for each pair of electron indices in a cluster C, a spin correlation $c_{ij}^{C}$ value can be calculated as the cluster average ${\langle \cdot \rangle }_{C}$

(9) $$c_{ij}^{C}=4\;{\langle m_{s,i}\;m_{s,j}\rangle }_{C}=\frac{4}{P_{C}}\sum_{R^{*}\in C}P_{R^{*}}\;m_{s,i}^{R^{*}}\;m_{s,j}^{R^{*}},$$

weighted by the probabilities $P_{R^{*}}$ and normalized by $4P_{C}$ to the interval [−1,+1]. Note that this definition is equivalent to the statistical correlation for singlet systems, because ${\langle m_{s,i}\rangle }_{C}=0$. Furthermore, the spin correlation is also equivalent to the identically named quantity in the Ising model [34].

We denote electron indices with $c_{ij}^{C}=-1$ (i.e., pairs in which the spins are always opposite) as ‘spin coupled’. Here, we define a group of spin-coupled electrons as an ‘electron motif’. If the motif contains more than two electrons, values of $c_{ij}^{C}=+1$ between next-neighboring electrons arise as a consequence. If one spin flips (comparing two maxima) in such an electron motif, all other spins of the motif flip as well.

**Figure A2:**
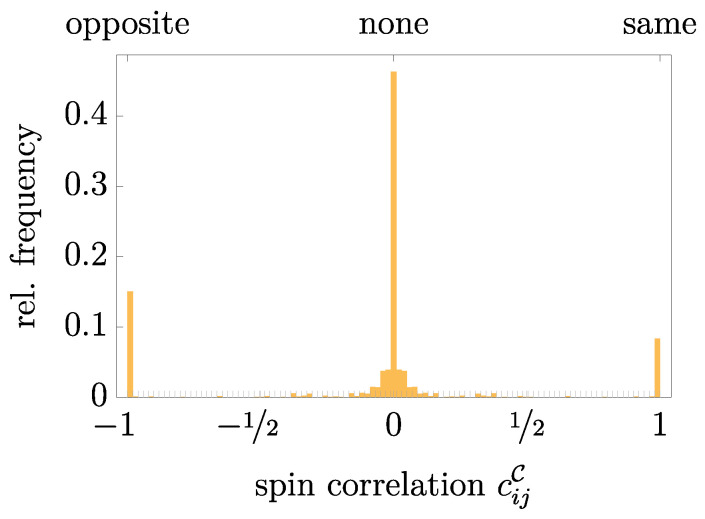
Histogram with 101 bins of spin-correlation values $c_{ij}^{C}$ calculated from all clusters of the molecules **1** to **29**. Same-spin correlation values ($c_{ij}^{C}=+1$) were only found within motifs composed of more than two electrons.

The distribution of spin-correlation values $c_{ij}^{C}$ over a range of molecules is displayed in Figure A2 in Appendix B and shows values of 0, −1, and +1 with much higher frequency compared to all other, intermediary values. The latter, partial spin-coupling values are closely and evenly distributed around $c_{ij}^{C}=0$ and will be subject of future works.

Finally, and in order to visualize the electron motif information obtained for a cluster in a condensed and comprehensible way, we visualize it in a representative maxima structure $R_{C}^{*}$ by black lines connecting the spin-coupled electrons (see Figure 1b for $H_{2}$ and Figure 2 for methanol). This finalizes the ab initio dot structure.

## 3. Computational Approach

### 3.1. Wave Function Generation

Throughout this work, we employed a correlated Slater-Jastrow wave function ansatz. This compact ansatz offers the explicit description of dynamic correlation [35]. Note that CAS and VB wave functions have recently been studied by two of the authors [12].

Gaussian 16 [36] was used for all density functional theory (DFT) calculations. All geometries were optimized with DFT (B3LYP [37,38,39] with the VWN(III) local correlation energy [40]) using the aug-cc-pVTZ basis [41]. For the subsequent single-point calculations, each function of the Slater-type triple-ζ TZPae basis [42] was expanded into 14 primitive Gaussian-type functions [43,44]. The Kohn-Sham orbitals for the Slater part of the wave function were obtained in this TZPae basis. Note that the basis set dependence of PDA has not yet been systematically investigated. This dependence is however—in principle—smaller than for non-real-space methods (e.g., valence bond theory), since the resulting wave function alone is investigated. Basis sets that give the same wave function also give the same PDA results.

Subsequently, an sm444 Schmidt-Moskowitz type Jastrow factor [35,45,46] was added to the Kohn-Sham determinant and energy minimized [47] with the open-source quantum Monte Carlo package *Amolqc* [48]. Together with the Slater-type basis set, this ensures a correct description of two-particle cusps [35,49].

### 3.2. Monte Carlo Integration of the Probability Density

The 3n-dimensional basin probability (3) integrals were calculated by Monte Carlo integration. A sample of size $N=10^{6}$ was drawn from the probability density p(R) for every investigated molecule during a standard variational Monte Carlo run [50].

For each sampled electron configuration R, p(R) is locally maximized to identify the basin $\Omega_{R^{*}}$, it is found in. The probability of a local maximum $R^{*}$ can then be calculated as

(10) $$P_{R^{*}}=\lim_{N\to \infty }\frac{N_{R^{*}}}{N},$$

where $N_{R^{*}}$ is the number of electron configurations found in the basin of attraction $\Omega_{R^{*}}$.

### 3.3. Local Maximization

Instead of maximizing *p*, −lnp is minimized. Note that −lnp(R) decreases monotonically with p(R). Therefore, it has the same basins and critical points as the probability density, but its minimization is more stable.

A two-phase minimization algorithm was employed. In the first phase (during a latency period), a step-length limited steepest descent algorithm is used to ensure the locality of the minimization. To avoid overshooting at the nuclear singularities, we employ a correction in the vicinity of nuclei [51]. The latency period is reset each time an electron arrives at a singularity. After the latency period, we smoothly switch to an L-BFGS-B algorithm [52], which moves the remaining valence electrons to their final positions in the maximum. The sampling of p(R), as well as the local minimization of the sampled electron configurations, were also conducted with *Amolqc* [48].

### 3.4. Clustering and Spin-Correlation Analysis

Clustering and spin-correlation analysis was conducted with the open-source software package *inPsights* [53].

The assignment problem (i.e., minimizing the cost function in (5)) is solved by the *Hungarian* algorithm [54] in cubic time $O(n^{3})$ [55]. If all *N* (potentially identical) maxima structures were compared with each other, the number of comparisons would grow with $O(N^{2})$. To allow for sample sizes of $N=10^{6}$, a pre-clustering strategy was employed to quickly reduce the number of structures $N^{\prime }\approx 10^{1}$ to $10^{3}$. For this, maxima were sorted by their function value and a greedy, spherical pre-clustering algorithm was employed with a sphere radius of $r=0.01a_{0}$. The algorithm is described in Appendix C. The maxima clustering calculations were conducted on a standard desktop computer with a single CPU. Computation times ranged from minutes to several hours depending on the number of electrons and the complexity of the ${\left|\Psi \right|}^{2}$ topology.

After the pre-clustering, we employed the DBSCAN clustering algorithm [56,57], where we set the minimal cluster size minPts=1 to conduct single-linkage clustering. The $d_{\mathrm{sim}}$ value was selected in the range of $0.1a_{0}$ to $0.4a_{0}$ with $0.2a_{0}$ being the default. Acetic acid and DHA were clustered locally. All other molecules (i.e., molecules shown in Table 1, Table 2, Table 3 and Table 4) have been clustered considering all electrons, and marginal probabilities were obtained by subsequent summation.

Infrequently, and for symmetrical molecules, spatially similar maxima are encountered that are exactly transformable into each other by rotation or reflection but for which a different distance-based assignment is found. If this different and arbitrary assignment leads to a spin flip, spin-correlation information is lost. In these cases, and to prevent this arbitrary assignment, the clustering $d_{\mathrm{sim}}$ was decreased, and the clusters were assigned manually.

In total, 31 molecules were investigated. Raw samples and maxima clustered data, and representative maxima are available in *binary*, *YAML*, and *x3dom HTML* format, respectively, in the supplementary material [58]. Because of file size limitations, only the first $2.5\times 10^{5}$ of the total $10^{6}$ sample points are available. The remaining data is available upon request.

## 4. Results and Discussion

First, a systematic study of the electron motifs found in the chemical bonds of small molecules including the elements H, B, C, N, O, and F is conducted. Second, we discuss the motif interaction in the carboxyl group of acetic acid. Lastly, the transferability of motifs is shown for the DHA molecule.

The probabilities presented are always local cluster probabilities for the depicted bond. These are either obtained directly from local clustering (cf. the discussion of methanol in Section 2.3) or by subsequent summation of cluster probabilities obtained from global clustering. Instead of the electron configuration of the representative maximum (cf. Figure 2), a schematic ab initio dot structure is shown for every cluster. Again, the electron positions are colored (blue and red) with respect to the $m_{s}$ quantum number. Yet, this coloring only has an implication within an electron motif of spin-coupled electrons (connected by black lines) but not between different electron motifs. For any depicted electron motif, all colors could be exchanged without changing the information of the structure.

### 4.1. Single Bonds

Small molecules exhibiting single bonds between the elements H, B, C, N, O, and F have been investigated. Based on the electron distribution among the two nuclei, covalent (**c**) and ionic structures can be identified and are represented by the ab initio dot structures in the Table 1 and Table 2, respectively. For the empty spots in the upper triangle of the Table 1, only ionic maxima are found, and vice versa.

For the series of homoatomic single bonds in dihydrogen (H−H), diborane(4) $D_{2d}$ ($H_{2}B-BH_{2}$), ethane ($H_{3}C-CH_{3}$), hydrazine ($H_{2}N-NH_{2}$), hydrogen peroxide (HO−OH), and difluorine (F−F), as expected, we find dominantly covalent structures **1c**, **7c**, **12c**, **16c**, **19c**, and **21c** with high probabilities. Only for hydrogen peroxide and difluorine, ionic structures (**19i**, **21i**) have a significant probability, as well. This strong ionic contribution may be related to the charge-shift character of these bonds, i.e., a large covalent–ionic resonance interaction energy in VB theory [59]. For heteroatomic single bonds, the probabilities of the ionic structures follow the trend which is expected, given the bond polarities derived from electronegativity (see the columns and rows of Table 2). Note that the dominance of the covalent structure is due to the correlated nature of the wave function. With a Hartree-Fock wave function, very similar electron positions are obtained for the homoatomic single bonds but with an independent distribution of the two opposite spin electrons on the two positions due to the lack of explicit Coulomb repulsion. Half the maxima, therefore, have two electrons at the same position, which is unphysical (except at the nuclei) and results in the typical ionicity of 50% in Hartree-Fock.

While all polarity trends are described well, a wrong bond polarity is found for a few molecules: for methane ($CH_{4}$) and ammonia ($NH_{3}$), hydridic (i.e., $H^{-}$) structures are found (**4i** and **5i**) but not the expected protonic (i.e., $H^{+}$) ones. This discrepancy is caused by the poor treatment of static correlation in the wave function ansatz. Earlier work [12] has shown that the probabilities are more reliable for VB and CAS wave functions. However, this work focuses on the shape of the structures, which is reliably predicted.

The dominant covalent structures of the single bonds are characterized by an electron pair shared between two atomic cores (see, for example, **12c** in Figure 1). The respective probability density maxima show one electron position outside each atomic core in direction to the other core. In contrast to the two core electron positions, this ‘bond position’ is displaced due to Pauli repulsion to the same-spin core electron and the Coulomb attraction to the second core (as discussed in detail in previous work [11]). Since hydrogen has no core electrons, the maximum position is always located directly at the nucleus, like in $H_{2}$ as discussed in Section 2.2. The electrons identified at the two bond positions always have opposite spin. Accordingly, the electron pair arises naturally as an electron motif.

Yet, the ab initio dot structures extend beyond the Lewis picture for several reasons. First, the ‘lone pair’ positions are all spin coupled for and cannot be divided into electron pairs without arbitrariness. Instead, they form intuitively understandable geometrical shapes avoiding other electrons (especially those of same spin) due to Coulomb and Pauli repulsion. Because they are all spin coupled, they form an electron motif that we will denote as ‘lone motif’ in the following. Second, there are single bonds, where a lone motif is additionally spin coupled to the bonding electron pair (**17c**, **18c**, **5c**, **14c**). Thus, there is a larger electron motif, which includes the lone positions, as well as the bond positions. However, the distinction between bond electrons and lone-pair electrons can still easily be made. Last, this is no longer the case for the minor covalent structures (**5c${}^{\prime }$**, **6c${}^{\prime }$**, **15c${}^{\prime \prime }$** and **18c${}^{\prime }$**) found mostly for bonds with fluorine. Here, an electron pair on the bond can no longer be identified. Instead, the border between bond and lone motif is blurred. These structures may be compared to inverted bonds discussed in VB theory [60,61].

In the following, individual covalent structures will be discussed in more detail. For hydrazine (**16c**), and similarly for methylamine ($H_{3}C-NH_{2}$, **13c**), and hydroxylamine ($H_{2}N-OH$, **17c**), the lone pair electrons are aligned with the depicted bond. For ammonia (**4c**), the lone pair is aligned to any of the three N−H bonds. For water (**5c**), hydroxylamine (**17c**), and hydrogen peroxide (**19c**), the lone motif is also coupled with the bond motif on the O−H bond, which is not explicitly shown and indicated by a dagger in Table 1. For water and methanol, the bond pair is tilted off the bond axis, which has already been observed by Scemama et al. [8].

In the difluorine molecule (**21c**), the lone motif at each atom arranges in a hexagon of alternating spins. In the depicted ab initio dot structure, the lone motif electron positions are not in one plane. Instead, the electrons with same spin to the next bond electron are always further away from the bond, which is easily explained with Pauli repulsion. However, this system is a good example to discuss the impact of the clustering threshold $d_{\mathrm{sim}}$: because of the discussed distortion of the hexagon, a spin coupling is predicted between the lone motif and the bond pair for very small $d_{\mathrm{sim}}$. However, we chose a value for $d_{\mathrm{sim}}$, which is comparable to that for other systems, and treat the distortion of the hexagon like the small displacements discussed for methanol (Figure A1 in Appendix A).

Other ab initio dot structures of single bonds can clearly be identified as ionic structures by the distribution of electrons to the cores (Table 2). For the homoatomic systems, only one of the two ionic structures is depicted. For all of the depicted dot structures, the ionic bond pair is always spin coupled to the lone motif and integrated into its geometry, forming a larger electron motif. Ionic fluorine structures (e.g., in hydrogen fluoride (H−F, **6i**) always show a cube motif, which is only slightly distorted and composed of two same-spin tetrahedrons. As discussed previously for the neon atom [11], this fits perfectly to both the cubic atom proposed by Lewis [1] and the double quartet theory by Linnett [7].

For first row atoms, there are two possible orientations for the ionic lone pair motifs: if an edge is directed towards the bond (e.g., for aminoborane ($H_{2}B-NH_{2}$, **9i**) and fluoromethane ($H_{3}C-F$, **15i**), a bond pair could still be identified. In this regard, the Lewis picture is recovered. This is no longer the case, if a vertex is directed towards the bond, e.g., for borinic acid ($H_{2}B-OH$, **10i**) and fluoroborane ($H_{2}B-F$, **11i**), as found only for boron- and hydrogen-containing bonds.

In summary, motifs found at single bonds are limited to at most three and exhibit clear structural regularities. Covalent structures are characterized by a bond pair that can be coupled to lone motifs, where individual electron pairs cannot be identified. In ionic structures, an additional pair of electrons is incorporated into a present lone motif. This will be different for multiple bonds, where increased spin-coupling and structural variety is observed.

### 4.2. Multiple Bonds

Small molecules exhibiting double and triple bonds between the elements C, N, and O have been investigated. Again, covalent and ionic structures are identified; see Table 3 and Table 4.

Whereas the position of the bond pair electrons could be rationalized for single bonds by a combination of Coulomb force and Pauli repulsion, additional electrons complicate the picture. In multiple bonds, there are two ways to accommodate these electrons. First, they can be added to the single bond structure, so that the bond pair motif stays relatively unchanged. Second, all electron positions could be moved from the bond axis, so that there are more equivalent bond positions. Whereas the former is related to the σ-π picture of multiple bonds, the latter fits to the τ picture, where the bond is decomposed into equivalent ‘banana bonds’. These equivalent bonds fit best with the Lewis dot structures and are also obtained as localized molecular orbitals with Edminston-Ruedenberg or Foster-Boys localization of molecular orbitals [62,63,64].

**Figure 3:**
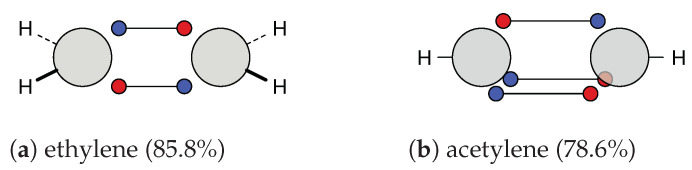
Ab initio dot structures of the most probable clusters of ethylene and acetylene with their cluster probabilities.

At first, the carbon-carbon bonds in ethylene ($H_{2}C=C_{2}H$) and acetylene (HC+CH) are discussed. For both systems, a covalent structure is dominant; see Figure 3.

As discussed in earlier work [11,12], these structures fit with the τ picture. This is no longer case the for the other systems in Table 3 and Table 4. To compare the double bond in ethylene to other double bonds, the main covalent cluster (Figure 3a) is divided into two clusters, so that the two two-electron motifs are replaced with a rectangular four-electron motif. This decomposition, which is again done only for comparison, leads to the two structures with alternating spins (**22c**) and blocked spins (**22c${}^{\prime }$**). The same is done for acetylene: the main covalent structure (Figure 3b) is decomposed into **27c** and **27c${}^{\prime }$**.

For ethylene, we find two symmetric, diagonal structures (**22c${}^{\prime \prime }$**) in addition to the one presented in Figure 3a. These structures are in good agreement with VB theory for τ bonds, which has already been discussed in earlier work [12]. Alternatively, they could be discussed as a modified single bond structure, where the additional electrons are placed close to the atoms and the single bond is only tilted off the bond axis.

For the double bond in methanimine ($H_{2}C=NH$), a rectangular bond motif (**23c**) is found without decomposition. It is coupled with the lone motif to an extended motif in which the spins are alternating. For nitroxyl (HN=O), a rectangular motif (**26c**) is also recovered, albeit distorted and also coupled with the lone motifs on both atoms. Formaldehyde ($H_{2}C=O$) is dominated by an ionic structure (**24i**) reflecting the polarity of the bond but shows a variation of the rectangular motif (**24c**) with two edges of a triangular prism of six electrons on O directed towards a pair of electrons from C as a covalent motif. In this structure, the identification of four bond electrons is no longer possible. Thus, in this aspect, it is comparable to the minor covalent structures (**c${}^{\prime }$**) found for single bonds.

The single structure for (E)-diimine (HN=NH, **25c**) can be related to both the rectangular motif and the diagonal motif: it can be described as a widened rectangle, to which a central bond motif has been added. Alternatively, it is a diagonal motif, to which to lone pairs have been added. Note that the observed spin coupling does not justify the latter. The minor covalent structure for methanimine (**23c${}^{\prime }$**) is a combination of the ethylene and the (E)-diimine structures **22c${}^{\prime }$** and **25c**. In formaldehyde, the second covalent motif (**24c${}^{\prime }$**) is best described as a tetrahedron of electrons between the atoms coupled to four lone pair electrons.

The ab initio dot structures of acetylene, dinitrogen (N≡N), and hydrogen cyanide (HC≡N) are compared in Table 4. The larger number of bond electrons leads to electron arrangements around the favorable maximum positions along the bond axis or to electrons at or near these positions with other spin-coupled electrons.

For the lone-pair free acetylene, regular, triangular prism motifs (**27c**, **27c${}^{\prime }$**) occur, when the dominant covalent structure (Figure 3b) is decomposed. Additionally, a triangular antiprism (**27c${}^{\prime \prime }$**) and a rectangular bond motif, extended by two additional electrons to a chair-like six-electron motif (**26${}^{\prime \prime \prime }$**), are found.

For hydrogen cyanide and dinitrogen, distorted (anti)prisms with one (**28c${}^{\prime }$**, **28c${}^{\prime \prime }$**) or two electrons (**29c${}^{\prime }$**, **29c${}^{\prime \prime }$**) at or near the single bond maximum positions along are observed, respectively. The dominant motif in dinitrogen (**29c**) has two electrons near the bond maximum positions forming the tips of two pyramids of alternating spin. The dominant motif in hydrogen cyanide (**28c**) is again a combination of the acetylene prism motif (**27c**) and the dominant dinitrogen motif (**29c**).

For hydrogen cyanide, a wedge-like ionic motif (**28i**) similar to that in formaldehyde (**24c**) in Table 3 is found. Again, the probability of finding the ionic motifs correlates with the electronegativity difference.

In summary, and as for the single bonds, a small variety of only three and four significant motifs are found per double and triple bond, respectively, for the investigated molecules. Furthermore, clear structural regularities and relationships could be identified, thus making them characteristic for the respective bond type. In contrast to the single bond motifs, the multiple bond motifs cannot be reconciled with the Lewis picture, as soon as lone pair electrons are involved. In this case, and with only one exception, all valence electrons are spin-coupled.

Overall, characteristic motifs were found for the two-center bonds investigated. Next, motif interactions at neighboring bonds are investigated at the example of acetic acid.

### 4.3. Motif Interaction in the Carboxyl Group of Acetic Acid

The electronic structure of molecules is predominantly local in the sense that functional groups exist with very similar properties in very different molecules. This locality is well described by the electron density for parts of the molecule [14], by localized molecular orbitals and by VB theory. Here, we can show that a many-electron view to locality is possible with the probability density analysis. In many cases, the ab initio dot structures found in small molecules containing a single functional group are directly transferable to molecules with several functional groups where they appear with comparable probabilities. In other cases, the structures are found with significantly altered probabilities, while some motifs or motif combinations are not found at all.

The carboxyl group (COOH) in acetic acid ($H_{3}C-COOH$) can be thought of as a hydroxy group (O−H) adjacent to a carbonyl group (C=O). Yet, chemically, the carboxyl group differs substantially from alcohols and ketones. A substantial alteration of the maxima is, therefore, expected. For the carbonyl group in acetic acid, we recover the ionic double bond motif of formaldehyde **24i** and the covalent double bond motif **24c${}^{\prime }$**, but we also find a distorted variant **24i${}^{\prime }$** of the ionic double bond motif **24i** with two electrons along the C=O bond axis and six electrons arranged in a hexagon around the O nucleus. This motif is known from fluoromethane (**15c**; see Table 1).

The ionic motifs are found with a probability of 49% for **24i** and 46% for **24i${}^{\prime }$** compared to 83% for **24i** in formaldehyde. The covalent motif **24c${}^{\prime }$** is recovered with 5% compared to 8% in formaldehyde. The covalent motif **24c** is found with <1% in acetic acid compared to 8% in formaldehyde.

**Figure 4:**
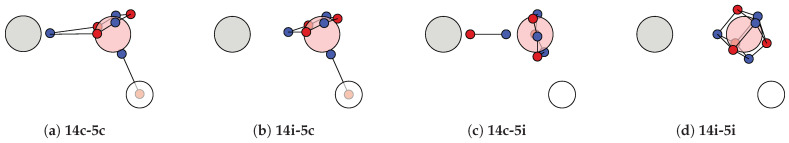
Combinations of C−O and O−H structures found at the C−O−H of acetic acid.

For the C−O−H group, the covalent and ionic C−O motifs (**14c** and **14i**) are identified, both combined with a covalent O−H bond motif (**5c**). In this context, these two C−O−H structures will be denoted **14c-5c** and **14i-5c**; see Figure 4a,b.

Two new structures arise for the C−O−H group in acetic acid, both with ionic O−H groups (i.e., with no electron at the proton). The first motif combines a covalent C−O part coupled with lone pairs and an ionic O−H part, denoted **14c-5i**, while the other is ionic both in C−O and O−H (**14i-5i**); see Figure 4c,d. The combined probability of finding **14c-5i** or **14i-5i** is 43% (see Table 5), while ionic O−H maxima are not found in water or methanol at all.

The substantially increased ionicity of the structures reflects the increased polarity of the O−H bond, and thus acidity, in acetic acid, compared to water and alcohols. A shift to ionic motifs is also found for the C−O group. The covalent C−O motif **14c** is found with 53% in methanol compared to 49% (**14c-5c** and **14c-5i**) in acetic acid and the ionic motif **14i** with 47% in methanol and with 51% in acetic acid (**14i-5c** and **14i-5i**); see Table 5.

In addition to new motifs found in the carboxyl group, we find that the C=O and C−O−H motifs are not statistically independent. We do not find a spin coupling to specific carboxyl motifs like the spin coupling of lone pair electrons to bond electrons but we do find strong statistical coupling between the motifs. In Table 5, all observed combinations of motifs and their probabilities are listed. The covalent C=O motif **24c${}^{\prime }$** is found only combined with the ionic C−O−H motif **14i-5c**, and the new motif **24i${}^{\prime }$** coexists only with the ionic C−O−H motifs **14i-5c** and **14i-5i**.

With PDA, the carboxyl group in acetic acid is obtained as a combination of C−O−H and C=O motifs, in which its probabilities are substantially altered in comparison with $H_{3}C-O-H$ and $H_{2}C=O$. The motif probabilities show a strong shift towards ionic motifs. Furthermore, the C−O−H and C=O motifs are not independent but strongly correlated. These findings correlate well with chemical and physical properties, such as acidity and infrared group frequencies of the carboxyl group, in comparison to free hydroxyl and ketone groups [65].

### 4.4. Motif Transferability in Dehydroalanine

**Figure 5:**
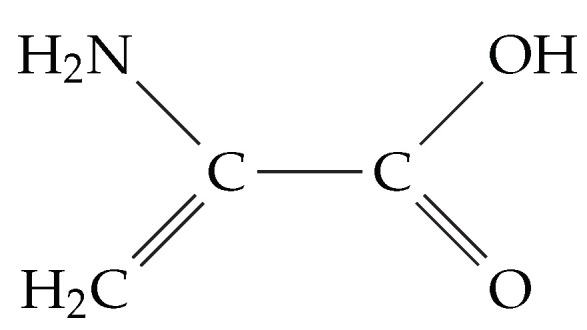
Dehydroalanine.

The DHA molecule depicted in Figure 5 contains a C=C double bond, a C−N single bond, and a carboxyl group.

The electron motifs of all three groups and their statistical correlations are investigated.

For DHA, the number of maxima and also the number of maxima clusters is substantially increased in comparison to the small molecules discussed above. However, it is not necessary to obtain and analyze all maxima clusters. Instead, we employ local clustering to identify the motifs and their probabilities for each functional group. Statistical correlations between neighboring functional groups can be analyzed by simultaneous local clustering of two functional groups. This way, large numbers of maxima clusters do not occur at any stage of the analysis, and the size of the molecules to be investigated is limited only by the possibility of running a variational Monte Carlo calculation.

For the individual functional groups, the same motifs are found as in the corresponding environments in the smaller molecules ethylene, methylamine, and acetic acid. In contrast to the discussion of the carboxyl group, no additional motifs arise. This indicates that the electronic motifs are indeed characteristic for functional groups.

Whereas the electron motifs of the smaller molecules are recovered in DHA, the individual probabilities of the motifs shift significantly. In Table 6, the probabilities for the motifs in DHA are compared with those of the corresponding smaller molecules.

These probability changes, once more, indicate an influence of the environment on the electronic structure in a functional group and deserve further investigation which is beyond the scope of this paper. Contrary to the situation in acetic acid, no statistical correlation between individual motifs of any two functional groups is observed.

## 5. Conclusions

In this work, the probability density analysis based on the local maxima of the squared wave function was presented. The existing analysis was expanded by a comprehensive clustering of these maxima, which allows for the definition of a spin correlation and the generalization of the electron pair to the electron motif. As an alternative to the empirical Lewis structures, as well as to the semi-empirical Linnett structures, ab initio dot structures have been introduced as the representation of a cluster and its spin information. These ab initio structures have been presented and analyzed for single, double, and triple bonds in small first row molecules. For single bonds, the ionic-covalent resonance of VB theory can be reproduced (covalent and ionic structures are identified), and the results mostly fit with the Lewis picture. However, the concept of distinct lone pairs cannot be reconciled with the findings by probability density analysis, so that they have been replaced with a lone motif. For multiple bonds, the agreement with the Lewis picture vanished for many systems, and extended electron motifs occur due to the coupling of the bond electrons with the lone motifs. In general, only very few motifs are found for each bond or functional group. In acetic acid, the motifs of the C−O−H group in methanol and the C=O group in formaldehyde are recovered, but additional motifs arise. Furthermore, the motifs of the two groups are strongly statistically correlated, reaffirming the status of the carboxyl function as an independent functional group from an electronic structure perspective. In DHA, on the other hand, the motifs of the three functional groups are very similar to the motifs of the corresponding smaller molecules, and no significant statistical correlation is found. This demonstrates the transferability of electron motifs corresponding to individual functional groups. Furthermore, the limited spatial extent of the motifs reflects the locality of chemistry at the level of real space many-electron analysis. This allows a probability density analysis for large molecules, in spite of the exponentially increasing number of local maxima, by employing a local clustering focused on only one or two bonds.

## Figures and Tables

**Table 1:** Covalent ab initio dot structures found for homo- and heteroatomic single bonds between the elements H, B, C, N, O, and F with their associated local cluster probabilities in parentheses. Motifs of spin-coupled electrons within are indicated by black lines.

	H	B	C	N	O	F
H	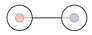 **1c**: H−H (96.5%)	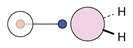 **2c**: $H-BH_{2}$ (91.4%)	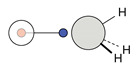 **3c**: $H-CH_{3}$ (97.4%)	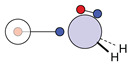 **4c**: $H-NH_{2}$ (99.5%)	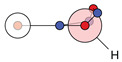 **5c**: H−OH (98.7%) ${}^{†}$	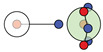 **6c**: H−F (43.9%)
B		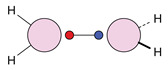 **7c**: $H_{2}B-BH_{2}$ (99.7%)	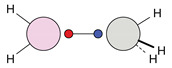 **8c**: $H_{2}B-CH_{3}$ (99.9%)		
C			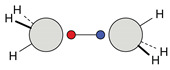 **12c**: $H_{3}C-CH_{3}$ (99.9%)	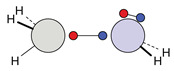 **13c**: $H_{3}C-NH_{2}$ (81.9%)	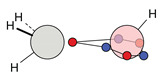 **14c**: $H_{3}C-OH$ (52.6%)	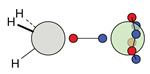 **15c**: $H_{3}C-F$ (0.3%)
N				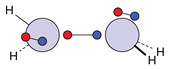 **16c**: $H_{2}N-NH_{2}$ (99.4%)	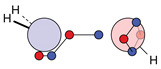 **17c**: $H_{2}N-OH$ (70.1%)	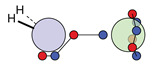 **18c**: $H_{2}N-F$ (53.1%)
O	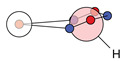 **5c${}^{\prime }$**: H−OH (1.1%) ${}^{†}$				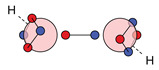 **19c**: HO−OH (60.6%) ${}^{†}$	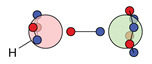 **20c**: HO−F (58.0%) ${}^{†}$
F	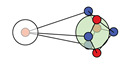 **6c${}^{\prime }$**: H−F (6.7%)		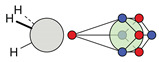 **15c${}^{\prime }$**: $H_{3}C-F$ (0.3%)	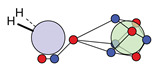 **18c${}^{\prime }$**: $H_{2}N-F$ (1.1%)		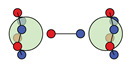 **21c**: F−F (60.3%)

† extended correlations with O−H electrons.

**Table 2:** Ionic ab initio dot structures found for homo- and heteroatomic single bonds between the elements H, B, C, N, O, and F with their associated local cluster probabilities in parentheses. Motifs of spin-coupled electrons within are indicated by black lines.

	H	B	C	N	O	F
H	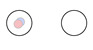 **1i**: H−H (2×1.8%)	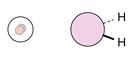 **2i**$H-BH_{2}$ (8.5%)	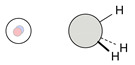 **3i**: $H-CH_{3}$ (2.1%)	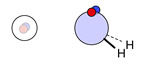 **4i**: $H-NH_{2}$ (0.5%)		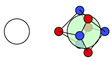 **6i**: H−F (49.4%)
B				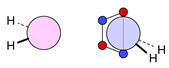 **9i**: $H_{2}B-NH_{2}$ (99.9%) ${}^{†}$	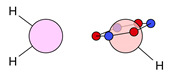 **10i**: $H_{2}B-OH$ (99.9%)	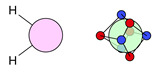 **11i**: $H_{2}B-F$ (100%)
C				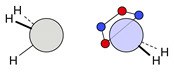 **13i**: $H_{3}C-NH_{2}$ (18.0%)	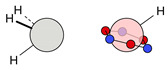 **14i**$H_{3}C-OH$ (47.4%)	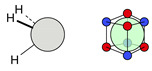 **15i**: $H_{3}C-F$ (99.3%)
N				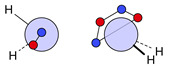 **16i**: $H_{2}N-NH_{2}$ (2×0.1%) ${}^{†}$	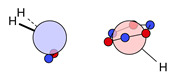 **17i**: $H_{2}N-OH$ (29.8%) ${}^{‡}$	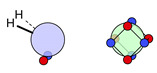 **18i**: $H_{2}N-F$ (45.9%)
O					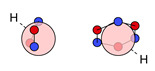 **19i**: HO−OH (2×18.7%)	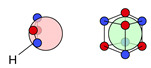 **20i**: HO−F (32.2%) ${}^{‡}$
F					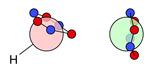 **20i’**: HO−F (9.8%)	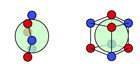 **21i**: F−F (2×19.9%)

† extended correlations with N−H electrons; ‡ extended correlations with O−H electrons.

**Table 3:** Covalent and ionic ab initio dot structures found for homo- and heteroatomic double bonds between the elements C, N, and O with their associated local cluster probabilities in parentheses. Motifs of spin-coupled electrons within are indicated by black lines.

	C	N	O
	**Covalent**		
C	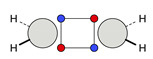 **22c**: $H_{2}C=CH_{2}$ (48.1%) ${}^{†}$	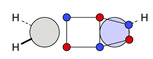 **23c**: $H_{2}C=NH$ (76.0%)	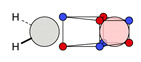 **24c**: $H_{2}C=O$ (8.3%)
N			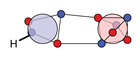 **26c**: HN=O (42.4%)
C	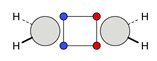 **22c${}^{\prime }$**: $H_{2}C=CH_{2}$ (37.7%) ${}^{†}$	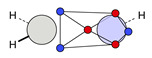 **23c${}^{\prime }$**: $H_{2}C=NH$ (23.8%)	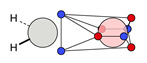 **24c${}^{\prime }$**: $H_{2}C=O$ (8.4%)
N		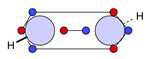 **25c**: HN=NH (100%)	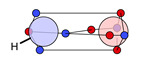 **26c${}^{\prime }$**: HN=O (20.5%)
C	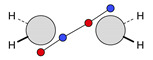 **22c${}^{\prime \prime }$**: $H_{2}C=CH_{2}$ (2×7.0%)		
	**Ionic**		
C		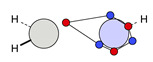 **23i**: $H_{2}C=NH$ (2×0.1%)	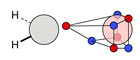 **24i**: $H_{2}C=O$ (2×41.3%)
N			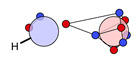 **26i**: HN=O (2×18.6%) ${}^{‡}$

† only for comparison, obtained by decomposition of the main covalent cluster; ‡ extended correlations with N−H electrons.

**Table 4:** Covalent and ionic ab initio dot structures found for homo- and heteroatomic triple bonds between the elements C and N with their associated local cluster probabilities in parentheses. Motifs of spin-coupled electrons within are indicated by black lines.

	C	N
	**Covalent**	
C	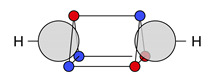 **27c**: HC≡CH (78.5%) ${}^{†}$	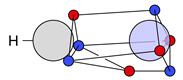 **28c**: HC≡N (72.8%)
N		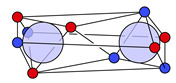 **29c**: N≡N (92.4%)
C	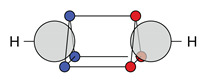 **27c${}^{\prime }$**: HC≡CH (0.1%) ${}^{†}$	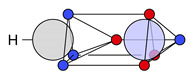 **28c${}^{\prime }$**: HC≡N (0.2%)
N		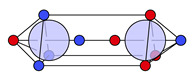 **29c${}^{\prime }$**: N≡N (0.7%)
C	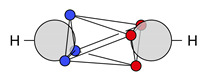 **27c${}^{\prime \prime }$**: HC≡CH (15.5%)	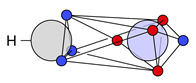 **28c${}^{\prime \prime }$**: HC≡N (5.9%)
N		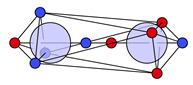 **29c${}^{\prime \prime }$**: N≡N (6.9%)
C	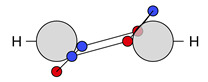 **27c${}^{\prime \prime \prime }$**: HC≡CH (5.8%)	
	**Ionic**	
C		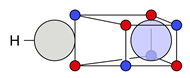 **28i**: HC≡N (20.9%)

† only for comparison, obtained by decomposition of the main covalent cluster.

**Table 5:** Local probabilities of all significant (P>1%) electronic motifs combinations in acetic acid.

C−O−H	C=O	$P_{\tilde{C}}$/%
**14i-5c**	**24i${}^{\prime }$**	30
**14c-5i**	**24i**	26
**14c-5c**	**24i**	22
**14i-5i**	**24i${}^{\prime }$**	16
**14i-5c**	**24c${}^{\prime }$**	4

**Table 6:** Local probabilities of motifs in dehydroalanine (DHA) compared to simple molecules (SM).

Group	Motif	$P_{\tilde{C}}\left(\mathrm{DHA}\right)$/%	$P_{\tilde{C}}\left(\mathrm{SM}\right)$/%
C=C	**22c** or **22c${}^{\prime }$**	93	86 ${}^{a}$
C=C	**22c${}^{\prime \prime }$**	6	14 ${}^{a}$
C−N	**13c**	73	82 ${}^{b}$
C−N	**13i**	27	18 ${}^{b}$
COOH	**14i-5c** and **24i${}^{\prime }$**	43	30 ${}^{c}$
COOH	**14c-5i** and **24i**	25	26 ${}^{c}$
COOH	**14c-5c** and **24i**	11	22 ${}^{c}$
COOH	**14i-5i** and **24i${}^{\prime }$**	19	16 ${}^{c}$
COOH	**14i-5c** and **24c${}^{\prime }$**	3	4 ${}^{c}$

^*a*^ ethene, ^*b*^ methylamine, ^*c*^ acetic acid.

## Data Availability

The data presented in this study are openly available in the Open Science Framework at 10.17605/OSF.IO/6Z2J9, reference number 6Z2J9.

## Appendix C. Pre-Clustering

To reduce the number of comparisons, we utilize that similar maxima also have similar probability density values p. Thus, by sorting the maxima and comparing only those within a window of values [−lnp,−lnp+Δ), the number of comparisons is often drastically reduced. We employ this in a spherical greedy pre-clustering algorithm, where we start with the first maximum as a cluster center and compare it with all maxima from the window. Maxima within a similarity sphere of radius *r* are then clustered together. The first maximum being outside of *r* constitutes the center of a new cluster sphere to which all subsequent maxima from the window must be compared. This is then repeated for all value windows. This algorithm is greedy because the creation of spherical cluster centers is random and depends on the order of maxima. As long as the sphere radius is chosen with $r\leq d_{\mathrm{sim}}/2$, the maxima in the pre-cluster spheres will be merged into the same clusters during the main-clustering, as they would have been without the pre-clustering step.
